# Machine Learning Approach to Decision Making for Insulin Initiation in Japanese Patients With Type 2 Diabetes (JDDM 58): Model Development and Validation Study

**DOI:** 10.2196/22148

**Published:** 2021-01-27

**Authors:** Kazuya Fujihara, Yasuhiro Matsubayashi, Mayuko Harada Yamada, Masahiko Yamamoto, Toshihiro Iizuka, Kosuke Miyamura, Yoshinori Hasegawa, Hiroshi Maegawa, Satoru Kodama, Tatsuya Yamazaki, Hirohito Sone

**Affiliations:** 1 Department of Internal Medicine Faculty of Medicine Niigata University Niigata Japan; 2 NTT Comware Corporation Tokyo Japan; 3 Department of Internal Medicine Shiga University of Medical Science Shiga Japan; 4 Faculty of Engineering Niigata University Niigata Japan

**Keywords:** hypoglycemic prescription, diabetes specialists, initial therapy, patterns of usage, machine learning

## Abstract

**Background:**

Applications of machine learning for the early detection of diseases for which a clear-cut diagnostic gold standard exists have been evaluated. However, little is known about the usefulness of machine learning approaches in the decision-making process for decisions such as insulin initiation by diabetes specialists for which no absolute standards exist in clinical settings.

**Objective:**

The objectives of this study were to examine the ability of machine learning models to predict insulin initiation by specialists and whether the machine learning approach could support decision making by general physicians for insulin initiation in patients with type 2 diabetes.

**Methods:**

Data from patients prescribed hypoglycemic agents from December 2009 to March 2015 were extracted from diabetes specialists’ registries, resulting in a sample size of 4860 patients who had received initial monotherapy with either insulin (n=293) or noninsulin (n=4567). Neural network output was insulin initiation ranging from 0 to 1 with a cutoff of >0.5 for the dichotomous classification. Accuracy, recall, and area under the receiver operating characteristic curve (AUC) were calculated to compare the ability of machine learning models to make decisions regarding insulin initiation to the decision-making ability of logistic regression and general physicians. By comparing the decision-making ability of machine learning and logistic regression to that of general physicians, 7 cases were chosen based on patient information as the gold standard based on the agreement of 8 of the 9 specialists.

**Results:**

The AUCs, accuracy, and recall of logistic regression were higher than those of machine learning (AUCs of 0.89-0.90 for logistic regression versus 0.67-0.74 for machine learning). When the examination was limited to cases receiving insulin, discrimination by machine learning was similar to that of logistic regression analysis (recall of 0.05-0.68 for logistic regression versus 0.11-0.52 for machine learning). Accuracies of logistic regression, a machine learning model (downsampling ratio of 1:8), and general physicians were 0.80, 0.70, and 0.66, respectively, for 43 randomly selected cases. For the 7 gold standard cases, the accuracies of logistic regression and the machine learning model were 1.00 and 0.86, respectively, with a downsampling ratio of 1:8, which were higher than the accuracy of general physicians (ie, 0.43).

**Conclusions:**

Although we found no superior performance of machine learning over logistic regression, machine learning had higher accuracy in prediction of insulin initiation than general physicians, defined by diabetes specialists’ choice of the gold standard. Further study is needed before the use of machine learning–based decision support systems for insulin initiation can be incorporated into clinical practice.

## Introduction

While oral antihyperglycemic agents are indicated for many patients with type 2 diabetes, some patients require insulin injections, with or without oral antihyperglycemic agents, in the advanced stages of diabetes. Since type 2 diabetes typically develops and progresses gradually and asymptomatically [1], it is often found at the first primary care consultation at a rather advanced stage with fatigue, thirst, and polyuria accompanied by substantially elevated plasma glucose levels. Such situations force physicians to judge whether to prescribe insulin as the initial therapy to avoid further disease progression. A physician’s misjudgment sometimes results in a hyperglycemic coma or another serious condition, as most patients hesitate to use insulin therapy because of inconvenience and cost [1,2]. Since there are no absolute standards for judgment of insulin initiation, this important decision made at the first consultation in primary care must be based on the physician’s knowledge of the pathophysiology of the patient’s condition and much prior experience. While diabetes specialists, defined as board-certified diabetologists, are trained on whether to choose insulin therapy based on their perception of the existence of complex conditions in their patients, as well as their overall health [3-5], such judgments are not easy for nonspecialists, defined as general physicians without board certification as diabetologists.

Machine learning, which can learn patterns and decision rules from data [6-9], has been used in clinical practice. Applications of machine learning for the early detection of diabetic retinopathy and cancer, for which clear-cut diagnostic gold standards exist, have been evaluated [10-16]. However, little is known about the usefulness of machine learning for decisions such as insulin initiation by specialists, for which there are no absolute criteria for use in clinical settings.

In this study, we first evaluated the ability of machine learning models to predict insulin initiation by specialists using the Japan Diabetes Clinical Data Management (JDDM) Study Group, which consists of diabetes specialists. Then, we compared the clinical decisions made by the machine learning approach (trained using the database of specialists’ judgments) with those made by nonspecialists regarding whether to prescribe insulin for patients with type 2 diabetes at the first consultation. Using this information, we attempted to clarify the ability of machine learning models and determine whether artificial intelligence might assist clinicians in deciding on the initial therapy for type 2 diabetes in clinical practice.

## Methods

### Study Participants

Data were extracted from patients prescribed hypoglycemic agents from December 2009 to March 2015 using software (CoDiC) developed by the JDDM Study Group to promote clinical research on diabetes. Details on the JDDM Study Group and CoDiC are described elsewhere [3,4,17,18]. Briefly, the JDDM Study Group is a large network of diabetes specialists in Japan in 98 facilities. Study participants were individuals aged 20 years or older who started medical treatment for type 2 diabetes in outpatient clinics. Of the 6864 participants who received initial monotherapy during the above time period, we excluded 2004 individuals because of missing data on covariates (age, sex, BMI, duration of diabetes, level of glycated hemoglobin [HbA_1c_], hypertension, and estimated glomerular ﬁltration rate [eGFR]). Thus, data were analyzed from 4860 patients who were prescribed antidiabetic medications including insulin as the initial medical treatment and had laboratory data (Table 1). The ethics committee of the JDDM Study Group and Niigata University approved this study (2012-7, 2017-0294). Informed consent was obtained from all patients at each participating institute in accordance with the Guidelines for Epidemiological Studies of the Ministry of Health, Labour and Welfare of Japan.

**Table 1:** Characteristics of study participants according to prescription of insulin or another hypoglycemic drug.

Characteristic		Insulin (n=293)	Noninsulin (n=4567)	*P* value
Age (years), mean (SD)		59 (14)	61 (13)	.009
**Age (years), n (%)**				
	<40	31 (11)	256 (6)	<.001
	40-59	114 (39)	1635 (36)	
	≥60	148 (51)	2676 (59)	
Male-to-female ratio		195:98	2929:1638	.40
BMI (kg/m^2^), mean (SD)		24.5 (4.2)	25.7 (4.6)	<.001
**BMI (kg/m^2^), n (%)**				
	<22.5	96 (33)	1045 (23)	<.001
	22.5-25.0	81 (28)	1158 (25)	
	≥25.0	116 (40)	2364 (52)	
Duration of diabetes (years), mean (SD)		9.2 (10.6)	6.8 (7.6)	<.001
**Duration of diabetes (years), n (%)**				
	<1.0 years	96 (33)	997 (22)	<.001
	1.0-9.9 years	89 (30)	2483 (54)	
	≥10.0 years	108 (37)	1087 (24)	
Hypertension, n (%)		138 (47)	2324 (51)	.23
Systolic blood pressure (mmHg), mean (SD)		132 (23)	131 (17)	.17
HbA_1c_^a^ (%) (NGSP)^b^, mean (SD)		8.8 (2.3)	7.6 (1.3)	<.001
**HbA_1c_ (%) (NGSP), n (%)**				
	<7.0	71 (24)	1444 (32)	<.001
	7.0-8.9	101 (34)	2498 (55)	
	≥9.0	121 (41)	625 (14)	
eGFR^c^ (mL/min/1.73 m^2^), mean (SD)		82.3 (31.4)	79.7 (21.0)	.16
**eGFR (mL/min/1.73 m^2^), n (%)**				
	<30	17 (6)	47 (1)	<.001
	30-59	45 (15)	617 (14)	
	≥60	231 (79)	3903 (85)	

^a^HbA_1c_: glycated hemoglobin.

^b^NGSP: National Glycohemoglobin Standardization Program.

^c^eGFR: estimated glomerular ﬁltration rate.

### Study 1

**Figure 1:**
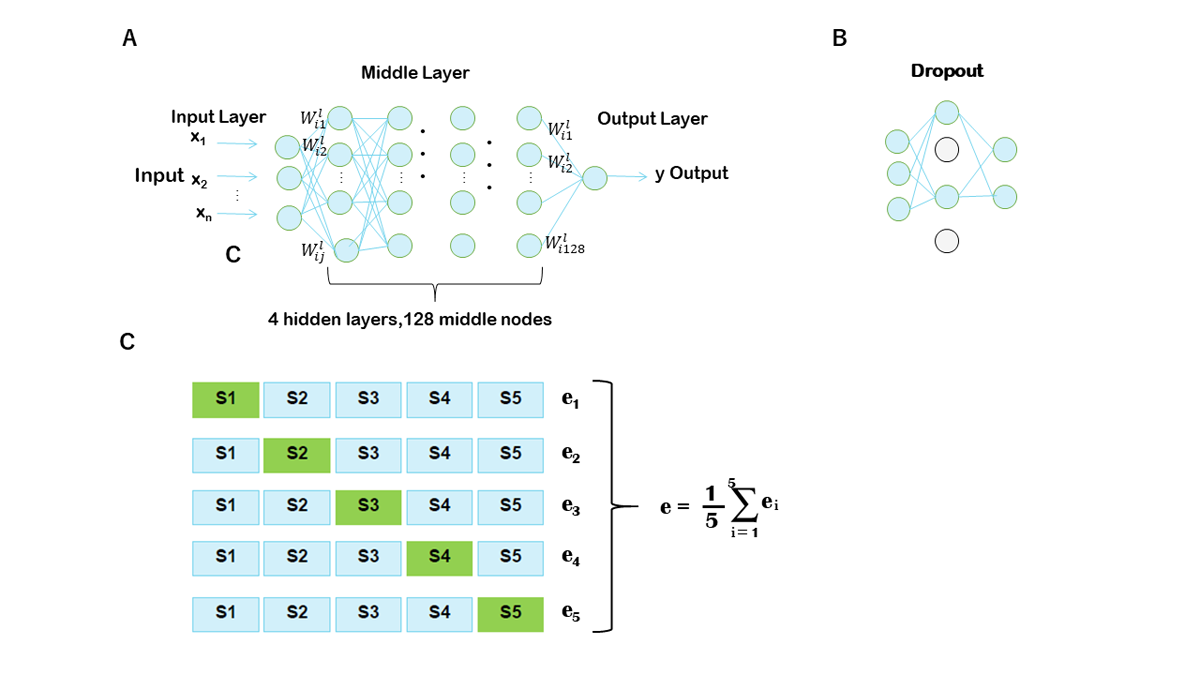
(A) Schematic diagram of our neural network models: X=(x1,…, xn) is the input vector and Y=y is the element of the output layer; Wlij is the weight between the ith neuron of the (l)th layer and the jth neuron of the (l-1)th layer . (B) Schematic diagram of dropouts. (C) Schematic diagram of 5-fold cross-validation; S1-S5 indicates data subsets 1 to 5.

We used the full JDDM Study Group data set (N=4860) to evaluate the ability of machine learning models with 5-fold cross-validation analysis for insulin initiation. We divided 4860 prescriptions into 5 groups, maintaining the noninsulin-to-insulin ratio within each group (overall noninsulin-to-insulin ratio of 4567:293). Each training set represented 80% of the data and each test set represented 20% (Figure 1C). We then performed random undersampling, and stratified extraction was adopted. The sampling ratio was verified after being changed to 1:2, 1:4, and 1:8. Specifically, first, using 4860 prescription patterns (ie, using no random undersampling data), the neural network was used to decide on the initial antihyperglycemic medication (insulin or noninsulin initiation). Similarly, using 2576 prescription patterns with a downsampling ratio of 1:2, 1434 prescription patterns with a downsampling ratio of 1:4, and 866 prescription patterns with a downsampling ratio of 1:8, the neural network was used to decide on the initial antihyperglycemic medication. Accuracy, recall, and area under the receiver operating characteristic (ROC) curves (AUCs) were calculated for insulin initiation. Accuracy was defined as the ratio of the sum of the true-positive and true-negative results for all cases. Recall was defined as the ratio of the true-positive cases to the sum of the true-positive and false-negative cases.

### Study 2

We compared clinical decisions made by the machine learning approach with those made using logistic regression and by general physicians as to whether to prescribe insulin for patients with type 2 diabetes at the first consultation. We used the full JDDM Study Group data set (N=4860). Forty-three cases that were randomly selected from the 4860 cases to be included in a questionnaire were used for validation data (Multimedia Appendix 1). In random undersampling, stratified extraction was adopted, and the sampling ratios were verified after being changed to 1:2, 1:4, and 1:8. Specifically, first, using 4817 prescription patterns (ie, using no random undersampling data), the neural network and logistic regression were used to decide on the initial antihyperglycemic medication (insulin or noninsulin initiation). Similarly, using 2545 prescription patterns with a downsampling ratio of 1:2, 1409 prescription patterns with a downsampling ratio of 1:4, and 841 prescription patterns with a downsampling ratio of 1:8, the neural network and logistic regression were used to decide on the initial antihyperglycemic medication. In the neural network, each training set represented 80% of the data. We repeated the training 5 times and calculated the average predictive value. The ability of the neural network and logistic analysis to predict insulin initiation in 43 patients was examined according to accuracy, recall, and AUCs.

### Study 3

We compared clinical decisions made by the machine learning approach and logistic regression with those made by nonspecialists regarding whether to prescribe insulin for patients with type 2 diabetes at the first consultation, focusing on more definitive cases. In study 3, we evaluated only 7 cases for which the choice of insulin as the initial antidiabetic medication was agreed upon by 8 of the 9 specialists who considered the 43 cases (Multimedia Appendix 2). The ability of a neural network and logistic analysis to predict insulin initiation was evaluated for accuracy.

### Questionnaires

This study used a questionnaire to compare the choice of the initiation of each antihyperglycemic drug between general physicians and specialists in clinical settings. We submitted the questionnaire to 50 physicians randomly selected from a list of general physicians (internal medicine physicians) without board certification as diabetologists in Niigata Prefecture; 22 general physicians completed the questionnaire. Nine specialists from university hospitals also completed the same questionnaire. Each physician chose the most suitable antidiabetic drug based on 7 variables (age, sex, BMI, duration of diabetes, HbA_1c_, hypertension, and eGFR) in 43 cases that were randomly selected from the JDDM Study Group database.

### Neural Networks

We used neural networks [19,20] to extract the choice of insulin use by diabetes specialists. A neural network is a mechanism of information processing that emulates the mechanisms of the brain to classify information and identify patterns. Figure 1A is a schematic diagram of our models, where X=(x_1_,…, x_n_) is the input vector, Y=y is the element of the output layer, and *W^l^_ij_* is the weight between the *i*th neuron of the (l)th layer and *j*th neuron of the (*l*-1)th layer. Seven explanatory variables (age, sex, BMI, duration of diabetes, HbA_1c_, hypertension, and eGFR) were used as input nodes (X1-X7), and the output was the predictive value of insulin use by the neural network. Figure 1C is an image of the cross-validation performed in study 1. For each test, 1 of the 5 subsets was used as the test set and the others were used as training sets. Then, the averages of accuracy, recall, and AUCs across all 5 trials were calculated (study 1). In study 2 and study 3, each training set represented 80% of the data. We repeated the training five times and calculated the average of the predictive value. In this study, because the number of patients who were prescribed insulin was relatively low, we used random undersampling [21,22] to alleviate the imbalance in the data. The numbers used in each random sampling were described above. We used 4 hidden layers, 128 middle nodes, and a rectified linear unit (Relu). Dropouts were also set to suppress overlearning (dropout rate: 0.2 for 1 layer; 0.5 for 2-4 layers) (Figure 1B) [23]. Overlearning was evaluated using learning curve analysis. The number of epochs was validated at 10,000 with the convergence of the difference between accuracy and loss in the learning process. The neural network output was “insulin use,” (ie, the predictive value, ranging from 0 to 1 with cutoffs of >0.3, >0.5, and >0.7 for the dichotomous classification of insulin use versus no insulin use in each analysis). The general physicians’ choices were compared with the predictions made by machine learning using the neural network for both 43 cases (study 2) and 7 cases (study 3).

### Laboratory Data and Definition of Hypertension

HbA_1c_ was converted from the Japanese Diabetes Society’s values into the National Glycohemoglobin Standardization Program’s equivalent values according to guidelines established by the Japan Diabetes Society [24]. eGFR was determined by an equation modiﬁed for the Japanese population as previously described [25]. Hypertension was defined as a systolic blood pressure ≥140 mmHg and/or a diastolic blood pressure ≥90 mmHg, or current use of antihypertensive agents.

### Statistical Analysis

Categorical variables were expressed as numerals and percentages and were compared with χ^2^ tests. Continuous variables were expressed as mean (SD) and were compared using the Student *t* test for comparisons within each group. Differences in accuracy between general physicians’ decisions and the decisions of logistic regression and machine learning were analyzed using the McNemar test. All statistical analyses were performed using SPSS software (version 19.0; IBM Corp) or Python programming. *P* values <.05 were considered statistically significant.

## Results

### Baseline Characteristics

Table 1 shows participants’ baseline characteristics. The number of participants receiving each treatment is shown in Multimedia Appendix 3. With the exceptions of sex, prevalence of hypertension, and systolic blood pressure, there were significant differences between the insulin and noninsulin groups. Participants who were prescribed insulin were younger and had lower BMIs, longer durations of diabetes, and worse glycemic control than those who were not prescribed insulin as their initial medication.

### Study 1

**Figure 2:**
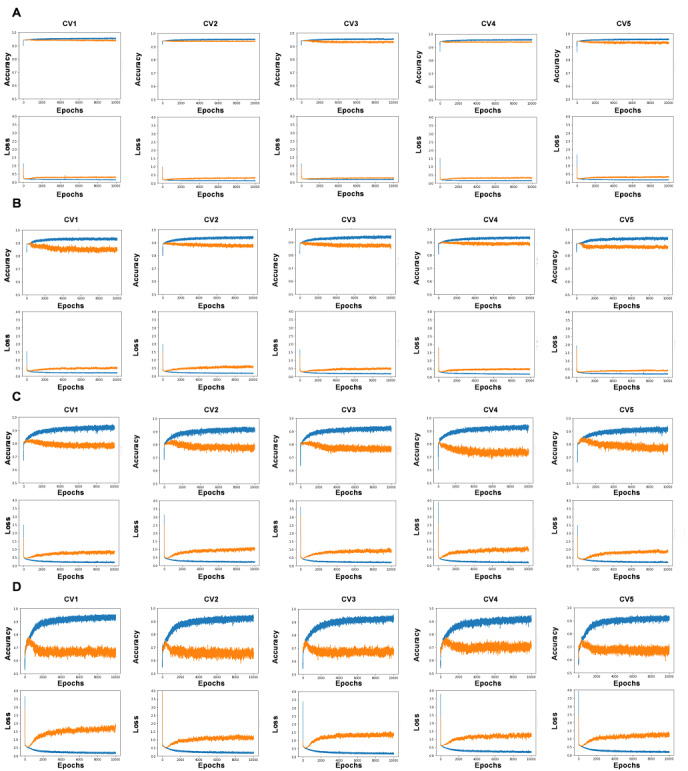
Learning curve analysis. (A) No undersampling. (B) Sampling ratio of 1:2. (C) Sampling ratio of 1:4. (D) Sampling ratio of 1:8. The top row shows the association between accuracy and number of epochs, and the bottom row shows the association between cross-entropy loss and number of epochs; the blue and orange lines show the results of training and validation, respectively. CV: cross-validation.

Table 2 shows the average accuracy, recall, and AUC of each neural network model using the full JDDM Study Group database (N=4860). Undersampling decreased accuracy but increased recall. AUCs for insulin initiation were approximately 0.6 to 0.7. In learning curve analysis, a tendency of overfitting was observed as the ratio of undersampling increased (Figure 2).

**Table 2:** Accuracy, recall, and area under the receiver operating characteristic curve (AUC) of each neural network model, with a cutoff of >0.5 for the dichotomous classification.

Cases	Accuracy	Recall	AUC
No undersampling	0.93	0.05	0.61
Sampling ratio 1:2	0.86	0.18	0.63
Sampling rato 1:4	0.78	0.34	0.69
Sampling rato 1:8	0.67	0.45	0.64

### Study 2

**Figure 3:**
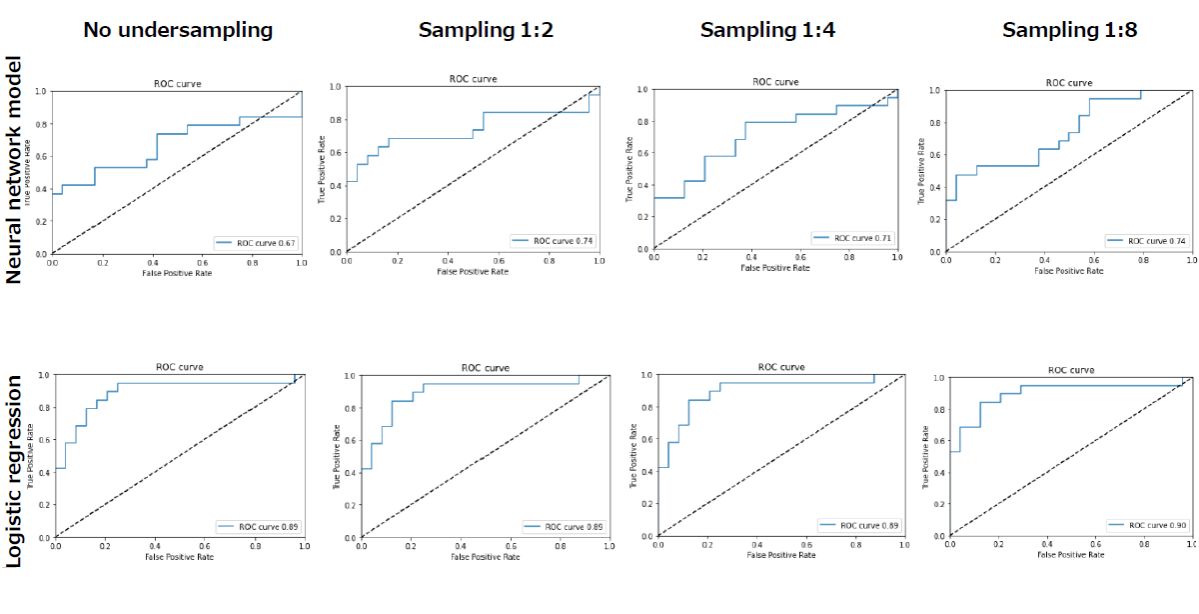
Receiver operating characteristic (ROC) curve of each neural network model and logistic regression for insulin initiation. The areas under the curve of the neural network model (upper row) for no undersampling, sampling ratio of 1:2, sampling ratio of 1:4, and sampling ratio of 1:8 were 0.67, 0.74, 0.71, and 0.74, respectively. For logistic regression (lower row), the areas under the curve for no undersampling, sampling ratio of 1:2, sampling ratio of 1:4, and sampling ratio of 1:8 were 0.89, 0.89, 0.89, and 0.90, respectively.

**Figure 4:**
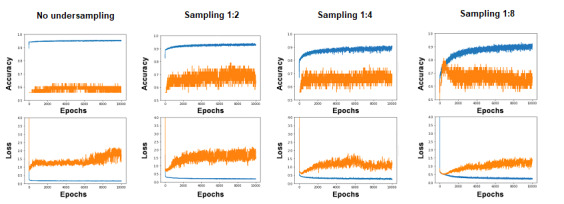
Learning curve analysis. (A) No undersampling. (B) Sampling ratio of 1:2. (C) Sampling ratio of 1:4. (D) Sampling ratio of 1:8. The top row shows the association between accuracy and number of epochs, and the bottom row shows the association between cross-entropy loss and number of epochs; the blue and orange lines show the results of training and validation, respectively.

Table 3 and Multimedia Appendices 4 and 5 show the accuracy and recall, and Figure 3 shows the ROC curves, of each neural network model and logistic regression in the 43 validation cases. The AUCs of the neural network models for no undersampling, sampling ratio of 1:2, sampling ratio of 1:4, and sampling ratio of 1:8 were 0.67, 0.74, 0.71, and 0.74, respectively, while the AUCs with logistic regression for no undersampling, sampling ratio of 1:2, sampling ratio of 1:4, and sampling ratio of 1:8 were 0.89, 0.89, 0.89, and 0.90, respectively. Accuracy and recall of logistic regression were higher than those of machine learning with a sampling ratio of 1:8. However, the difference in accuracy between the decisions made by logistic regression and machine learning was not statistically significant. Figure 4 shows the learning curve analysis. A tendency of overfitting was observed as the ratio of undersampling increased. The overall accuracy and recall of general physicians were 0.60 and 0.16, respectively. The difference in accuracy between logistic regression and general physicians was statistically significant with a cutoff of >0.5 for the dichotomous classification in the sampling ratio of 1:8 (*P*<0.05). We found no statistical significance between machine learning and general physicians.

**Table 3:** Accuracy and recall of each neural network model and logistic regression with a cutoff of >0.5 for the dichotomous classification.

Models		Accuracy	Recall
**Neural network model**			
	No undersampling	0.60	0.11
	Sampling ratio 1:2	0.72	0.37
	Sampling ratio 1:4	0.65	0.37
	Sampling ratio 1:8	0.70	0.52
**Logistic regression**			
	No undersampling	0.58	0.05
	Sampling ratio 1:2	0.65	0.21
	Sampling ratio 1:4	0.67	0.26
	Sampling ratio 1:8	0.81	0.68

### Study 3

The overall accuracy of insulin initiation by general physicians was 0.51 for the 7 cases for which the choice of insulin as the initial antidiabetic medication was agreed upon by 8 of the 9 specialists. The average predictive values (output) of insulin initiation by machine learning were 0.18, 0.40, 0.50, and 0.82, respectively, for no undersampling and sampling ratios of 1:2, 1:4, and 1:8 (Multimedia Appendix 6). The average predictive values for insulin initiation by logistic regression were 0.38, 0.52, 0.66, and 0.80, respectively, for no undersampling and sampling ratios of 1:2, 1:4, and 1:8 (Multimedia Appendix 6). The accuracies of logistic regression and the machine learning model using 0.5 for the dichotomous classification were 1.00 and 0.86, respectively, with a downsampling ratio of 1:8, which were higher than the accuracy of the general physicians (ie, 0.43) using 50% for the dichotomous classification.

## Discussion

### Principal Findings

To the best of our knowledge, despite its preliminary stage, this is the first trial to determine whether important clinical decisions, such as the selection of antidiabetic medication, made by a machine learning system could be comparable with decisions made by diabetes specialists or general physicians. Although we found no superior performance of machine learning over logistic regression, recall in machine learning was relatively similar to that of logistic regression analysis (study 2). In study 3, the accuracy of machine learning with a sampling ratio of 1:8 was higher than that of general physicians. Although further study is needed before machine learning–based decision support systems can be used for insulin initiation in clinical practice, these findings suggest the possibility that machine learning may support such decisions by general physicians.

Barnes et al [26] revealed that models using 7 variables (eg, age, family history of diabetes, BMI, fasting venous glucose level, HbA_1c_, prior gestational diabetes mellitus, and early diagnosis of gestational diabetes mellitus) could predict required insulin therapy with the addition of medical nutrition therapy in women with gestational diabetes. They showed that the AUC for the prediction of insulin use was 0.71 [26], a value similar to that found in our neural network model. In our study, logistic regression analysis using 7 variables showed that the accuracy and AUC for initial insulin/noninsulin discrimination were consistently higher than with the neural network. A review by Christodoulou et al [27] showed that evidence was lacking to support the claim that clinical prediction models based on machine learning lead to better AUCs than those based on logistic regression. Stylianou et al [28] revealed that an established logistic regression model performed as well as more complex machine learning methods in predictions of mortality from burns. Although recall in machine learning was relatively similar to that of logistic regression analysis in our study, further study is needed before machine learning can be used for decisions on insulin initiation in clinical practice because the neural network model cannot be clearly explained.

In our study, accuracy and recall in logistic regression with a cutoff of >0.3 for the dichotomous classification were higher than with a cutoff of >0.5 although this trend was not observed in the neural network model (Multimedia Appendix 3). Recall was modestly decreased in the neural network model with a cutoff of >0.7 for the dichotomous classification compared with the model with a cutoff of >0.5 for the dichotomous classification. Those findings suggest that with the neural network models, recall might be reduced even with a relatively high cutoff value as a discriminating criterion. However, although insulin initiation is an important clinical decision, recall was relatively low in our neural network model. Therefore, this issue of recall should be resolved before using machine learning–based decision support systems for insulin initiation in clinical practice.

We used random undersampling because the number of patients who were prescribed insulin was relatively low. Also, we attempted to reduce overlearning using dropouts. However, overfitting was still present, especially with the undersampling ratio of 1:8. Thus, no conclusions can be drawn on the usefulness of machine learning as a support system for decisions on insulin initiation until these issues are addressed.

Shortcomings in the accuracy of the prediction of insulin initiation may result from the influence of areas of ambiguity in our study, as there are no absolute standards for insulin initiation. This is in contrast to cancer imaging, for example, where there are consistent gold standards. In summary, the final decision depends on each physician. In fact, predicting insulin initiation through the use of only 7 clinical variables was a limitation mandated by lack of more complete data on our cohort. Predictability of insulin initiation could have been significantly improved if baseline information were available on the symptoms of hyperglycemia, weight loss, metabolic decompensation and ketosis, time course and severity of hyperglycemic symptoms, comorbidities, cardiovascular disease, microvascular complications, dementia, mental disorders, and various results of blood tests, such as C-peptide and glutamic acid decarboxylase antibody. These are key factors in the choice of insulin as initial treatment. Lyons et al [29] showed that initial body weight and peak insulin response were able to predict whether insulin therapy would be required in the subsequent 6 years in symptomatic diabetic patients aged 40 to 60 years with newly diagnosed diabetes. Moreover, doctor and patient values and preferences should be considered in the choice of antihyperglycemic drugs [2]. Since our findings are at a preliminary stage, further studies are needed to produce a tool to support decision making using machine learning in clinical practice, including aspects related to both doctors and patients.

As shown by Case D in Multimedia Appendix 6, machine learning could not predict insulin initiation. The duration of diabetes in Case D was only 0.2 years, which suggests that glucose metabolism worsened in a relatively short period of time. Diabetes specialists choose insulin as the initial therapy to prevent acute exacerbation of glycemic control. Therefore, the findings in Case D indicate that specific cases should be treated with insulin therapy regardless of other clinical variables.

Although we randomly selected a cohort of 43 patients to evaluate the predictability of machine learning, those 43 patients had a lower mean BMI and HbA_1c_ level compared with the entire patient sample (N=4860). The percentages of initial prescriptions of insulin differed between the entire cohort and the 43 randomly selected patients, leading to a discrepancy in the rate of insulin initiation between these two cohorts. Moreover, the insulin-to-noninsulin ratio was not strictly consistent with previous reports [4,19]. In addition, we selected 7 cases as the gold standard based on agreement of 8 of the 9 specialists in study 3. However, the number of validation samples was too small to conclude the usefulness of the ability of machine learning to predict insulin initiation.

The 7 variables in our study were those frequently encountered in clinical settings [4,5]; however, both general physicians and specialists may be unaware that all of these 7 factors could play a role in decisions regarding the use of insulin. Therefore, a simple, automatic, electronic medical system might be useful in addressing this problem. Unfortunately, our findings could not establish the cutoff levels for some variables, such as age, duration of diabetes, BMI, and eGFR, because of the small sample size. Further studies are needed to establish a meaningful decision-making support tool for use in actual consultations with regard to precision medicine.

### Study Limitations

Our study has several limitations. First, we randomly selected only 43 samples from the JDDM Study Group database for our questionnaire, as general physicians and specialists are reluctant to respond to long questionnaires. Therefore, the insulin-to-noninsulin ratio was not consistent with that observed by general physicians in clinical settings. Second, although we tried to reduce overlearning using cross-validation and dropouts, overfitting was still present. Thus, our findings should be interpreted with caution. Third, we could not obtain certain information, such as weight loss and hyperglycemic symptoms, that would affect insulin prescriptions because of incomplete data in the CoDiC database. In addition, selection bias was a concern because we included only patients with type 2 diabetes with data available on all 7 variables. In any case, our findings are at a preliminary stage and future studies are needed to produce a decision-making support tool for machine learning in clinical settings that includes those important variables. Fourth, the fact that the study population was exclusively ethnic Japanese may limit wider applicability of the results. Fifth, all of the gold standard cases were males.

### Conclusion

Although we found no superior performance of machine learning over logistic regression, machine learning had higher accuracy in the prediction of insulin initiation than general physicians, defined by diabetes specialists’ choice of the gold standard. Further study is needed before machine learning–based decision support systems for insulin initiation can be introduced into clinical practice.

## Appendix

Multimedia Appendix 1 Characteristics of study participants in the cohort of 43 patients.

Multimedia Appendix 2 Characteristics of study participants for which initial use of insulin was agreed upon by 8 of 9 specialists.

Multimedia Appendix 3 Number of patients receiving each hypoglycemia agent.

Multimedia Appendix 4 Accuracy and recall of each neural network model and logistic regression.

Multimedia Appendix 5 Accuracy and recall of each neural network model and logistic regression.

Multimedia Appendix 6 Accuracy and predictive value of each of 7 study participants for insulin initiation by neural network, logistic regression, and general physicians.

## Abbreviations

AUC: area under the receiver operating characteristic curve
eGFR: estimated glomerular ﬁltration rate
HbA1c: glycated hemoglobin
JDDM: Japan Diabetes Clinical Data Management
ROC: receiver operating characteristic
