# Supplementary material for: Machine Learning Approach to Decision Making for Insulin Initiation in Japanese Patients With Type 2 Diabetes (JDDM 58): Model Development and Validation Study
Source: JMIR Med Inform. 2021 Jan 27;9(1):e22148. doi: 10.2196/22148 (PMC7875702; doi:10.2196/22148)
Supplement: Multimedia Appendix 1 [file medinform_v9i1e22148_app1.docx]

**Supplemental Table 1.** Characteristics of study participants in the cohort of 43 patients.

| **Characteristics** | entire cohort | 43-patient cohort | *p* value |
| --- | --- | --- | --- |
| Number of patients (*n*) | 4817 | 43 |  |
| Age (y) | 61 ± 12 | 65 ± 11 | 0.024 |
| <40 | 287 (6) | 3 (7) | 0.542 |
| 40-59 | 1749 (36) | 8 (18) |  |
| ≥60 | 2824 (58) | 32 (74) |  |
| Male/female | 15 / 3 | 31 / 12 | 0.147 |
| Body mass index (kg/m^2^) | 25.7 ± 4.6 | 23.6 ± 4.1 | 0.024 |
| <22.5 | 1082 (22) | 18 (42) | 0.058 |
| 22.5-25 | 59 (1) | 10 (23) |  |
| ≥25 | 3719 (78) | 15 (35) |  |
| Duration of diabetes (years) | 7.0 ± 7.8 | 11.3 ± 9.7 | 0.350 |
| <1 year | 1093 (22) | 6 (14) | 0.302 |
| 1-9.9 year | 2572 (53) | 19 (44) |  |
| ≥10 years | 1195 (25) | 18 (42) |  |
| Hypertension, *n* (%) | 9 (57) | 20 (47) | 0.468 |
| HbA1c (%) (NGSP) | 7.7 ± 1.4 | 8.3 ± 2.4 | 0.001 |
| <7.0 | 1515 (31) | 11 (26) | <0.001 |
| 7.0-8.9 | 2599 (53) | 22 (51) |  |
| ≥9.0 | 746 (15) | 10 (23) |  |
| eGFR (mL/min/1.73m^2^) | 79.8 ± 21.8 | 80.1 ± 28.0 | 0.072 |
| <30 | 64 (1) | 2 (5) | 0.058 |
| 30-59 | 662 (14) | 4 (9) |  |
| ≥60 | 4134 (85) | 37 (86) |  |

NGSP, National Glycohemoglobin Standardization Program; eGFR, estimated glomerular ﬁltration rate
