# Supplementary material for: Machine Learning Approach to Decision Making for Insulin Initiation in Japanese Patients With Type 2 Diabetes (JDDM 58): Model Development and Validation Study
Source: JMIR Med Inform. 2021 Jan 27;9(1):e22148. doi: 10.2196/22148 (PMC7875702; doi:10.2196/22148)
Supplement: Multimedia Appendix 2 [file medinform_v9i1e22148_app2.docx]

**Supplemental Table 2.** Characteristics of study participants for which initial use of insulin was agreed upon by 80% of specialists.

|  | Age (year) | Sex | Duration of diabetes (year) | BMI (kg/m^2^) | HbA1c (%) | HT | eGFR (mL/min/1.73m^2^) |
| --- | --- | --- | --- | --- | --- | --- | --- |
| A | 64 | Male | 30.6 | 20.4 | 10.2 | + | 19.3 |
| B | 63 | Male | 12.7 | 21.7 | 11.5 | - | 93.1 |
| C | 65 | male | 17.2 | 20.1 | 11.3 | + | 140.2 |
| D | 57 | male | 0.2 | 23.1 | 14.0 | - | 67.4 |
| E | 64 | male | 20.8 | 15.5 | 8.9 | + | 137.5 |
| F | 64 | male | 8.9 | 19.7 | 13.8 | - | 86.9 |
| G | 66 | male | 18.8 | 14.9 | 17.2 | - | 158.3 |

BMI, body mass index; HT, hypertension; eGFR, estimated glomerular ﬁltration rate
