# Supplementary material for: Machine Learning Approach to Decision Making for Insulin Initiation in Japanese Patients With Type 2 Diabetes (JDDM 58): Model Development and Validation Study
Source: JMIR Med Inform. 2021 Jan 27;9(1):e22148. doi: 10.2196/22148 (PMC7875702; doi:10.2196/22148)
Supplement: Multimedia Appendix 3 [file medinform_v9i1e22148_app3.docx]

**Supplemental Table 3.** Number of patients receiving each hypoglycemia agent.

| Number of patients (*n*) | 4860 |
| --- | --- |
| Insulin | 293 |
| Sulfonylureas | 306 |
| Biguanides | 1152 |
| DPP-4 inhibitors | 2261 |
| α-Glucosidase inhibitors | 390 |
| Glinides | 191 |
| Thiazolidinediones | 111 |
| Sodium-glucose co-transporter 2 inhibitors | 97 |
| Glucagon-like peptide-1 receptor agonists | 159 |
