# Supplementary material for: Machine Learning Approach to Decision Making for Insulin Initiation in Japanese Patients With Type 2 Diabetes (JDDM 58): Model Development and Validation Study
Source: JMIR Med Inform. 2021 Jan 27;9(1):e22148. doi: 10.2196/22148 (PMC7875702; doi:10.2196/22148)
Supplement: Multimedia Appendix 4 [file medinform_v9i1e22148_app4.docx]

**Supplemental Table 4.** Accuracy and recall of each neural network model and logistic regression.

|  | Accuracy | Recall |
| --- | --- | --- |
| Neural network model |  |  |
| No under sampling | 0.63 | 0.16 |
| Sampling ratio 1:2 | 0.74 | 0.47 |
| Sampling ratio 1:4 | 0.63 | 0.42 |
| Sampling ratio 1:8 | 0.60 | 0.57 |
| Logistic regression |  |  |
| No under sampling | 0.65 | 0.21 |
| Sampling ratio 1:2 | 0.74 | 0.42 |
| Sampling ratio 1:4 | 0.81 | 0.68 |
| Sampling ratio 1:8 | 0.84 | 0.84 |

Cutoff >0.3 for the dichotomous classification
