# Supplementary material for: Machine Learning Approach to Decision Making for Insulin Initiation in Japanese Patients With Type 2 Diabetes (JDDM 58): Model Development and Validation Study
Source: JMIR Med Inform. 2021 Jan 27;9(1):e22148. doi: 10.2196/22148 (PMC7875702; doi:10.2196/22148)
Supplement: Multimedia Appendix 6 [file medinform_v9i1e22148_app6.docx]

**Supplemental Table 6.** Accuracy and predictive value of each of 7 study participants for insulin initiation by neural network, logistic regression and general physicians.

|  | General physicians | Neural Network | | | | Logistic regression | | | |
| --- | --- | --- | --- | --- | --- | --- | --- | --- | --- |
| Case |  | Predictive  no under  sampling | Predictive value  sampling ratio 1:2 | Predictive value  sampling ratio 1:4 | Predictive value  sampling ratio 1:8 | Predictive  no under  sampling | Predictive value  sampling ratio 1:2 | Predictive value  sampling ratio1:4 | Predictive value  sampling ratio1:8 |
| A | 0.59 | 0.73 | 0.96 | 0.99 | 1.00 | 0.42 | 0.58 | 0.74 | 0.85 |
| B | 0.36 | 0.19 | 0.52 | 0.82 | 0.86 | 0.19 | 0.33 | 0.48 | 0.70 |
| C | 0.41 | 0.02 | 0.04 | 0.01 | 0.79 | 0.14 | 0.25 | 0.38 | 0.60 |
| D | 0.45 | 0.08 | 0.60 | 0.17 | 0.20 | 0.45 | 0.63 | 0.77 | 0.88 |
| E | 0.18 | 0.00 | 0.00 | 0.00 | 0.87 | 0.78 | 0.88 | 0.93 | 0.98 |
| F | 0.64 | 0.21 | 0.42 | 0.96 | 0.99 | 0.24 | 0.39 | 0.56 | 0.73 |
| G | 0.95 | 0.00 | 0.23 | 0.55 | 1.00 | 0.41 | 0.58 | 0.74 | 0.86 |

a cutoff of >0.5 for the dichotomous classification
